# On the use of the outcome variable “small for gestational age” when gestational age is a potential mediator: a maternal asthma perspective

**DOI:** 10.1186/s12874-017-0444-z

**Published:** 2017-12-11

**Authors:** Geneviève Lefebvre, Mariia Samoilenko

**Affiliations:** 1 Department of Mathematics, Université du Québec à Montréal, C.P. 8888, Succursale Centre-ville, Montréal, Québec H3C 3P8 Canada; 2 Faculty of Pharmacy, Université de Montréal, Montréal, Canada

## Abstract

**Background:**

The variable “small for gestational age,” frequently defined as birth weight below the 10th percentile in a gestational age and sex-normalized population, is nowadays generally perceived as a more adequate measure than birth weight or low birth weight (birth weight < 2500 g) to capture fetal growth. However, the use of small for gestational age rather than birth weight or low birth weight as an outcome (dependent) variable may have important impacts on the interpretation of analyses aimed at estimating the causal effect of an exposure of interest on infants. We hypothesized potential differences in both types of effects estimated (direct or total) and in ability to control for confounding bias.

**Methods:**

We first examined the use of outcome variables birth weight and small for gestational age to get insights on modeling practices within the field of maternal asthma. Using directed acyclic graph simulations where gestational age was a potential mediator, we then compared estimated exposure effects in regression models for birth weight, low birth weight, and small for gestational age. Graphs with and without confounding were considered.

**Results:**

Our simulations showed that the variable small for gestational age captures the direct effect of exposure on birth weight, but not the indirect effect of exposure on birth weight through gestational age. Interestingly, exposure effect estimates from small for gestational age models were found unbiased whenever exposure effect estimates from birth weight models were affected by collider bias due to conditioning on gestational age in the models.

**Conclusions:**

The sole consideration of the outcome small for gestational age in a study may lead to suboptimal understanding and quantification of the underlying effect of an exposure on birth weight-related measures. Instead, our results suggest that both outcome variables (low) birth weight and small for gestational age should minimally be considered in studies investigating perinatal outcomes.

## Background

Perinatal outcomes birth weight (BW) and low birth weight (LBW; BW < 2500 g) have a long history of use in public health and medical studies [1]. Since the past several decades, there has been an increased awareness and understanding of the limitation of these variables to convey notions of prematurity and fetal growth [1–3]. Although no numeric cut-offs were proposed at that time, the concept of being “small for gestational age” (SGA) can be traced back to the 1960s [1]. Nowadays, the variable SGA, frequently defined as BW below the 10th percentile in a gestational age (GA) and sex-normalized population [4, 5], is generally accepted as a more adequate measure than BW or LBW to characterize intrauterine growth [2, 6]. Indeed, (L)BW can be viewed as a heterogeneous variable influenced by two distinct processes, GA and fetal growth, which can complicate the interpretation of study results. However, while much of recent focus is put on the epidemiology of preterm birth and SGA (e.g., [1, 7]), the use of SGA rather than (L)BW itself as an outcome (dependent) variable may have analytical consequences which can pose difficulties if they are not well known or understood.

In this work we have investigated the practical implications, both in terms of types of estimated effects and potential biases, of using different birth weight-related parametrizations (that is either (L)BW or SGA) as outcome variables when assessing the effect of exposures in analyses which use these outcomes. As mentioned previously, the rationale for not exclusively using (L)BW in studies is now well understood. Although SGA is perceived as a more interpretable outcome than (L)BW since internally adjusted for GA, it remains that this variable is a mere statistical construct (e.g., [8]) and could also present some limitations.

Direct and indirect exposure effects are intuitive concepts which are at the heart of mediation analyses [9–11]. Fundamentally, one often desires to decompose the total effect of an exposure on an outcome in one effect that is mediated by an intermediate variable (indirect effect) and one effect that does not arise through that variable (direct effect). The potential for GA to lie in the causal pathway between an exposure of interest (e.g., inhaled corticosteroids (ICS) for treating asthmatic pregnant women) and BW is clearly evident. Indeed, any effect of the exposure on GA necessarily entails an indirect effect of the exposure on BW because of the strong causal association between GA and BW. Using mediation ideas, we have conceived a simulation study with the goal to shed further light on the advantages and disadvantages of considering either (L)BW or SGA as outcome variable of interest in exposure effect analyses. Our objective is to emphasize the statistical implications of using standard modelling approaches for variables that could formally be cast into mediation models.

Using directed acyclic graphs (DAGs) [12, 13] where GA is a potential mediator between the exposure to ICS and BW, we first examined different scenarios wherein the effect of ICS on the outcome was either direct, indirect or both. Because SGA is strictly a function of BW and GA, we then assessed the corresponding interpretations for this outcome. Using mediation DAGs with confounders, we also examined different scenarios which could bias similarly or dissimilarly the estimates of the exposure-(L)BW and exposure-SGA associations. To our knowledge, no comprehensive simulation-based study has yet brought evidence concerning the differential implications of using either parametrization in the presence of confounding biases.

The paper is divided as follows. We first offer a glance at practice in maternal asthma research with respect to the use of (L)BW, SGA and GA as outcome variables. While one can argue that these are relatively crude measures of fetal and subsequent infant health, these outcomes are widely used in this specific research area. Then we describe the two sets of simulated scenarios considered, the processes used to generate the data, and the analyses performed. The presentation of the results and a discussion conclude this work.

## Method

### A glance at practice in maternal asthma research

To get insights about the choice of (L)BW and SGA as outcome variables of interest, we selected two relatively recent articles which performed a meta-analysis or a systematic review on populations of asthmatic pregnant women. Our selection is not deemed exhaustive but rather insightful of preferred habits in the use of (L)BW and SGA as outcome variables in maternal asthma research.

**Fig. 1:**
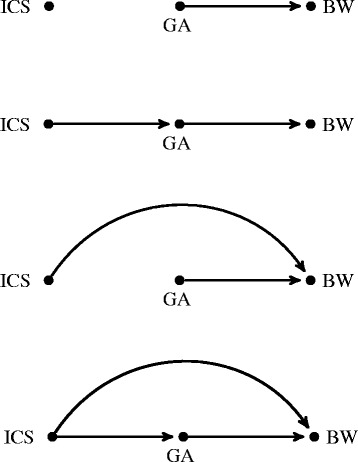
Directed acyclic graphs for the Basic Scenarios 1–4. From top to bottom, Basic Scenario 1: Null effect of ICS on BW; Basic Scenario 2: Indirect effect of ICS on BW; Basic Scenario 3: Direct effect of ICS on BW; Basic Scenario 4: Direct and indirect effects of ICS on BW

The first article, authored by Murphy et al. [14], presents a meta-analysis of adverse perinatal outcomes in women with asthma. The meta-analysis is based on cohort studies published between 1975 and 2009 and for which the effect of asthma was assessed for at least one of the following outcomes: LBW, SGA, and preterm birth (PTB; GA < 37 weeks). Table 1 presents the selection of these three outcomes by included study; this table was constructed from the studies reported in relative risk Figures 1–3 in Murphy et al. [14].

**Table 1:** Meta-analysis by Murphy et al. [14]: a summary of selected outcome variables by included study

Study	Publication dateª	LBW	SGA	PTB
Lao et al. [30]	1990	X	−	X
Perlow et al. [31]	1992	X	X	X
Doucette et al. [32]	1993	X	−	X
Jana et al. [33]	1995	X	−	X
Schatz et al. [34]	1995	X	X	X
Stenius-Aarniala et al. [35]	1995	−	−	X
Demissie et al. [36]	1998	X	X	X
Liu et al. [37]	2001	−	X	X
Bracken et al. [38]	2003	−	X	X
Dombrowski et al. [39]	2004	−	X	X
Acs et al. [40]	2005	X	−	X
Bakhireva et al. [41]	2005	−	X	X
Sheiner et al. [42]	2005	X	−	−
Clark et al. [43]	2007	−	X	−
Enriquez et al. [44]	2007	−	X	−
Kallen et al. [45]	2007	X	X	X
Karimi et al. [46]	2008	X	−	X
Breton et al. [47]	2009	X	X	X

*Abbreviations: LBW* low birth weight, *PTB* preterm birth, *SGA* small for gestational age

ª Presented by increasing order of publication date

The second article, by Eltonsy et al. [15], presents a systematic review for the use of *β*_2_-agonists during pregnancy and their effects on perinatal outcomes. This systematic review searched for articles published before 2013 regarding the effects of *β*_2_-agonists on congenital malformations, SGA, mean and low BW, GA, and PTB. Table 2 presents the selection of the three types of outcomes investigated by included study; this table was constructed from the studies reported in Tables 4–8 in Eltonsy et al. [15].

**Table 2:** Systematic review by Eltonsy et al. [15]: summary of selected outcome variables by included study

Study	Publication dateª	(L)BW	SGA	PTB∕GA
Schatz et al. [48]	1988	X	X	X
Lao et al. [15]^b^	1990	X	−	X
Schatz et al. [49]	1997	X	X	−
Alexander et al. [50]	1998	X	−	X
Olesen et al. [51]	2001	X	−	X
Braken et al. [23]^b^	2003	−	X	X
Schatz et al. [52]	2004	X	X	X
Bakhireva et al. [26]^b^	2005	X	X	X
Clifton et al. [53]	2006	X	X	X
Clark et al. [28]^b^	2007	X	X	−

*Abbreviations: GA* gestational age, *(L)BW* (low) birth weight, *PTB* preterm birth, *SGA* small for gestational age

ª Presented by increasing order of publication date

^b^ Study listed in Table 1

We see that the GA-related variables (GA, PTB) most often appear in the studies listed in Tables 1 and 2. Thus, when only two variables were considered in a study, it usually included a GA-related variable. In such a situation, the other covariate selected was seen to split in a larger proportion for (L)BW as opposed to SGA (8/12 vs 4/12, respectively; studies counted once only). In the meta-analysis results (see Table 1), 5 out of 18 studies (27.8%) reported all three outcomes, and 4 out of 10 (40%) for the systematic review (see Table 2). Three studies, all in the meta-analysis from Murphy et al. [14], only reported either LBW or SGA (without GA).

No clear time trend with respect to the patterns of inclusion of the variables is seen in Tables 1 and 2. More recent articles also show differential preferences regarding the use of variables (L)BW, SGA, and GA/PTB. For instance, in [16] and [17], the authors used all these three variables as outcomes in analyses, while [18] and [19] only used SGA and GA, and LBW and GA, respectively. Although the use of (L)BW without SGA (and vice versa) is not widespread based on these articles and the selected meta-analysis and systematic review, it is frequent enough to establish the relevance of investigating (L)BW and SGA jointly.

In the next section, we introduce a series of DAGs to help interpret estimated causal effects of an exposure on (L)BW and SGA. At the same time, we also incorporate the GA dimension since the constructed variable SGA is a function of both BW and GA.

### Design of primary simulations

We first present basic scenarios and corresponding data generation processes. These were used to gain insights on the interpretation of the associations between the exposure and the outcomes of interest ((L)BW, SGA). Then, to investigate the potential for confounding or collider bias [20, 21] when using (L)BW or SGA, we pursue with more complex scenarios that incorporate a single confounder of the relationships between the exposure, GA, and BW. Collider bias can be described as a spurious association between the exposure and the outcome which arises when studies, at the design or the analysis stage, stratify or adjust on a collider; in a DAG, a collider is a common effect of two variables lying on a path linking the exposure and the outcome [11]. The simulations were performed using R [22], version 3.0.2. We used the same initial seed to generate the data for all scenarios.

#### Basic scenarios

We considered four basic causal DAGs with three nodes: the exposure node ICS, the mediator node GA, and the outcome node BW. Figure 1 presents the DAGs showing the posited links between ICS, GA, and BW. Each of the DAGs corresponds to one scenario and is the basis for the generation of the corresponding variables of interest. First, a causal link between GA and BW is assumed in all four scenarios. Basic Scenario 1 depicts the case where ICS has no effect on BW, either direct or indirect. Basic Scenario 2 is a scenario in which the effect of ICS on BW is fully mediated by GA; in other words, the effect of ICS on BW exclusively occurs through a modification in GA. Basic Scenario 3 represents the situation where ICS only has a direct effect on BW; in this case, the effect of ICS on BW does not occur through a modification in GA. Finally, in Basic Scenario 4, ICS has a direct effect on BW, in addition to having an indirect effect mediated through GA. It can be noted that there are two scenarios which do not feature GA as a mediator of the relationship between ICS and BW (Basic Scenarios 1 and 3). These were considered as a benchmark for interpreting the results under scenarios in which GA is a mediator (Basic Scenarios 2 and 4).

#### Generation of outcomes (L)BW and SGA (basic scenarios)

For each basic scenario, we simulated data for 20,000 babies, where this process was repeated 1000 times to constitute 1000 samples of size 20,000. Exposures to ICS were generated from Bernoulli experiments with probability 0.5 (ICS = 1 if exposed and ICS = 0 if unexposed).

For each sample in the basic scenarios, the GAs for all babies were initially generated from a multinomial distribution with support between 26 and 43 weeks and probabilities corresponding to the estimated probability of delivering at each of these weeks according to Table 1 from Kramer et al. [4]. More precisely, the GAs were generated with theoretical probabilities coinciding with the empirical probabilities found in that table (that is, number of pregnancies observed for a given GA value divided by the total number of pregnancies). To generate the GAs for exposed babies in scenarios in which ICS had a direct effect on GA (Basic Scenarios 2 and 4), we subtracted 2 weeks from the GAs that were generated in the first place. The magnitude of this effect was chosen to well illustrate the concepts described herein and is likely larger than the real effect of ICS on GA. Indeed, low-to-moderate doses of ICS are generally regarded as safe regarding GA- and BW-related outcomes while some uncertainty remains regarding the effects of larger doses of ICS [23].

The BWs (in grams) were then generated independently according to a normal distribution with mean

1 $$ {\mu}_{BW}=-3703.3+183.25\  GA+{\beta}_{ICS}\  ICS $$

and standard deviation *σ*_*BW*_ = 333.82. The value of *β*_*ICS*_ in Eq. (1) was set to −100 or 0, depending on whether ICS had a direct effect on BW or not. The values of the intercept and GA coefficients were defined on the basis of the data found in Table 1 from Kramer et al. [4]. Specifically, these two coefficients had been *a priori* calculated by fitting a linear regression model for BW versus GA on a large simulated sample (*n* = 100,000), where the GAs had also been generated according to tabulated empirical probabilities and the BWs generated from a normal distribution according to the GA-specific mean and standard deviation values found in that table. The value 333.82 for *σ*_*BW*_ corresponds to the average of the standard deviation values found in the table and is substantially smaller than the residual standard error returned by the aforementioned large sample regression analysis (450.80). This value for *σ*_*BW*_ was selected so that less variability is observed for the BW distributions conditional on the smallest GA values. Although the assumptions of a linear effect of GA on BW and of common variance of errors are not satisfied in the data summarized in Table 1 from Kramer et al. [4], these were made in the primary simulations for simplicity.

We created the SGA variables for all babies based on their values for GA and BW. The binary variable SGA was determined by comparing a baby’s BW to the 10th percentile of the normal BW distribution conditional on GA. For example, an unexposed baby born at 28 weeks of gestation was found small for his GA (SGA = 1) if his BW was less than 999.89 g and not small for his GA otherwise (SGA = 0), where 999.89 is the 10th percentile of a normal distribution with mean equal to −3703.3 + 183.25 × 28 = 1427.7 and standard deviation equal to 333.82. An exposed baby born at 28 weeks of gestation was also said small for his GA if his BW was less than 999.89 g. A common BW threshold was thus used to determine the SGA value of every baby born at the same GA. Finally, a baby was said having LBW if his BW was smaller than 2500 g.

#### Confounding scenarios

**Fig. 2:**
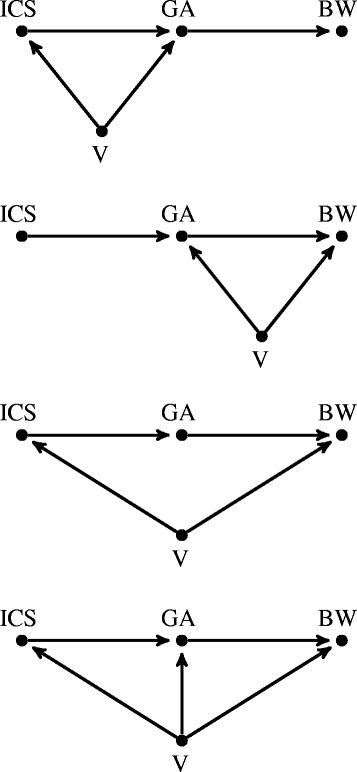
Directed acyclic graphs for the Confounding Scenarios 1–4. From top to bottom, Confounding Scenario 1: Indirect effect of ICS on BW with V confounder between ICS and GA; Confounding Scenario 2: Indirect effect of ICS on BW with V confounder between GA and BW; Confounding Scenario 3: Indirect effect of ICS on BW with V confounder between ICS and BW; Confounding Scenario 4: Indirect effect of ICS on BW with V common confounder between ICS, GA, and BW

Next, we considered four additional DAGs with four nodes: a dichotomous confounder node V and the same three nodes as before (ICS, GA, BW). Figure 2 presents the DAGs depicting the causal links between V, ICS, GA, and BW in these confounding scenarios. All the DAGs feature a causal effect of ICS on BW fully mediated by GA. The DAGs differ by the posited relationships between V and the nodes ICS, GA, and BW. In Confounding Scenario 1, V is a confounder between ICS and GA, while V is a confounder between GA and BW in Confounding Scenario 2. In Confounding Scenario 3, V is a confounder between ICS and BW. Confounding Scenario 4 encompasses all previous scenarios as V is a common cause of ICS, GA, and BW simultaneously.

#### Generation of outcomes (L)BW and SGA (confounding scenarios)

We also generated 1000 samples according to each of the four confounding scenarios. Each sample included 20,000 babies, among which half (10,000) had the value V = 1 and half had the value V = 0. Whenever there was an arrow from V to ICS in a DAG (Confounding Scenarios 1, 3, 4), the probability of being exposed to ICS was set to 0.7 for those with V = 1 and to 0.3 for those with V = 0. Otherwise (Confounding Scenario 2), the probability of being exposed to ICS was 0.5, independently of the value of V. Exposures to ICS were then generated according to these probabilities. Of note, the former values of 0.70 and 0.30 were selected to induce significant confounding arising through covariate V and exposure ICS. In general, in our simulations, we have allowed for strong relationships between variables to more comprehensively interpret the results.

For each sample, the GAs for the babies with V = 0 were generated from the multinomial distribution previously described. In Confounding Scenario 3, in which V had no direct effect on GA, the generated GA values for the babies with V = 1 were taken to be the same as those for the babies with V = 0. These values constituted the intermediate GA values for all babies. To create the GAs for the babies with V = 1 in scenarios in which V had a direct effect on GA, we subtracted 2 weeks from the GAs that were generated for the babies with V = 0. Lastly, to generate the final GA values for the exposed babies in scenarios in which ICS had a direct effect on GA, we subtracted 2 weeks from each exposed baby’s intermediate GA. For all other babies, their intermediate GA value was set to their final GA value.

The BWs (in grams) were generated independently according to a normal distribution with mean

2 $$ {\mu}_{BW}=-3703.3+183.25\  GA+{\beta}_{ICS}\  ICS+{\beta}_V\ V $$

and standard deviation *σ*_*BW*_ = 333.82. The value of *β*_*ICS*_ (*β*_*V*_) in Eq. (2) was equal to - 100 or 0, depending on whether ICS (V) had a direct effect on BW or not. The variables SGA and LBW were created as in the Basic Scenarios 1–4.

### Primary analyses (all scenarios)

On each sample generated according to each of the four *basic scenarios*, we fitted four linear or logistic models (*M*), depending or SGA was modeled:

$$ {M}_1:\kern0.5em E\left[ BW\right]={\beta}_0+{\beta}_{ICS}\  ICS; $$

$$ {M}_2:\kern0.5em E\left[ BW\right]={\beta}_0+{\beta}_{ICS}\  ICS+{\beta}_{GA}\  GA; $$

$$ {M}_3:\mathrm{logit}\left(P\left( LBW=1\right)\right)={\beta}_0+{\beta}_{ICS}\  ICS; $$

$$ {M}_4:\mathrm{logit}\left(P\left( SGA=1\right)\right)={\beta}_0+{\beta}_{ICS}\  ICS. $$

For each model under each basic scenario, we computed the mean of the 1000 estimated ICS coefficients, ${\widehat{\beta}}_{ICS}$, where each coefficient represents either a mean difference or a log odds ratio. The standard error of the mean of ${\widehat{\beta}}_{ICS}$ was calculated as the standard deviation of the 1000 estimated ICS coefficients divided by $\sqrt{1000}$.We also assessed whether the Monte Carlo 95% normal confidence interval for *β*_*ICS*_ included zero.

Similarly, we fitted the following eight models on each sample generated according to each of the four *confounding scenarios*:

$$ {M}_1:\kern1.25em E\left[ BW\right]={\beta}_0+{\beta}_{ICS}\  ICS; $$

$$ {M}_{1,V}:\kern0.75em E\left[ BW\right]={\beta}_0+{\beta}_{ICS}\  ICS+{\beta}_V\ V; $$

$$ {M}_2:\kern1.5em E\left[ BW\right]={\beta}_0+{\beta}_{ICS}\  ICS+{\beta}_{GA}\  GA; $$

$$ {M}_{2,V}:\kern0.75em E\left[ BW\right]={\beta}_0+{\beta}_{ICS}\  ICS+{\beta}_{GA}\  GA+{\beta}_V\ V; $$

$$ {M}_3:\kern1.25em \mathrm{logit}\left(P\left( LBW=1\right)\right)={\beta}_0+{\beta}_{ICS}\  ICS; $$

$$ {M}_{3,V}:\kern0.75em \mathrm{logit}\left(P\left( LBW=1\right)\right)={\beta}_0+{\beta}_{ICS}\  ICS+{\beta}_V\ V; $$

$$ {M}_4:\kern1.25em \mathrm{logit}\left(P\left( SGA=1\right)\right)={\beta}_0+{\beta}_{ICS}\  ICS; $$

$$ {M}_{4,V}:\kern0.75em \mathrm{logit}\left(P\left( SGA=1\right)\right)={\beta}_0+{\beta}_{ICS}\  ICS+{\beta}_V\ V. $$

We calculated the mean of the 1000 estimates ${\widehat{\beta}}_{ICS}$ obtained for each model under each confounding scenario, with corresponding standard error. Again, we assessed whether the confidence interval for *β*_*ICS*_ included zero. As an aid for interpretation, we also indicated whether the estimators of the ICS effect on (L)BW (${\widehat{\beta}}_{ICS}$ in models *M*_1 − 3_, *M*_1 − 3, *V*_) are biased according to the DAGs considered (e.g., see Pearl [24] for graphical causal rules). Whether or not the estimators of the ICS effect on SGA (${\widehat{\beta}}_{ICS}$ in models *M*_4_, and *M*_4, *V*_) are biased was also determined; in this case our insight relied on the estimates obtained from the simulations under the unconfounded basic scenarios.

### Sensitivity analyses (all scenarios)

In the primary simulations, we made the assumptions of a linear effect of GA on BW and of common variance of BW errors. We subsequently decided to create additional simulation scenarios to examine the consequence of fitting the standard regression models previously described when these assumptions were not verified in the data.

We assessed the potential impact of the heterogeneity of BW errors on the basic and confounding scenarios’ results. Instead of simulating the BWs with constant errors (*σ*_*BW*_ = 333.82 for all GA), we simulated them according to the GA-specific standard deviation values found in our perinatal reference table (that is, Table 1 from Kramer et al. [4]). For instance, we used *σ*_*BW*_ = 241.00 for a baby born at 28 weeks and *σ*_*BW*_ = 447.00 for a baby born at 40 weeks. Modification to the way that the SGA variable was created was done accordingly. More precisely, the 10th percentile cut-off at a given GA was determined using the GA-specific standard deviation value used for simulating the BWs at that GA. No changes were made regarding the linearity of the effect of GA on BW in these new analyses; that is, Eq. (1) and (2) still apply for describing the mean BW formula used for generating this outcome.

We also assessed the potential impact of the nonlinearity of the effect of GA on BW on the basic and confounding scenarios’ results. To do that, we took an approach similar to what was done to calculate the intercept and GA coefficient values in Eq. (1). However, instead of fitting a simple linear model on the large sample with GA as a single explanatory variable, we considered a model with additional quadratic and cubic GA terms. The coefficients returned by this model were used to specify the equations used to generate the BW values for these sensitivity analyses. For both the basic scenarios and the confounding scenarios with nonlinearity of GA effect on BW, we thus had

$$ {\mu}_{BW}=29415-2922.4\  GA+94.46\ {GA}^2-0.9378\ {GA}^3+\varphi \left( ICS,V\right) $$

where *φ*(*ICS*, *V*) is the last term in Eq. (1) for the basic scenarios, and *φ*(*ICS*, *V*) is the sum of the last two terms in Eq. (2) for the confounding scenarios; the standard deviation of errors remained the same at *σ*_*BW*_ = 333.82. For these analyses, we also fitted either one or two additional models $\left({M}_2^{\ast },{M}_{2,V}^{\ast}\right)$ where polynomial terms in GA up to degree 3 were included as regressors.

## Results

### Primary analysis results

The results for the basic and confounding scenarios for the primary simulations are presented in Tables 3 and 4, respectively.

**Table 3:** Basic scenarios without confounding

Scenario (model, interpretation of effect)	mean	se	null ICS effect?
Basic 1 (*M*_1_, total)	−0.2374	0.2025	yes
Basic 1 (*M*_2_, direct)	−0.0152	0.1441	yes
Basic 1 (*M*_3_, total)	0.0046	0.0027	yes
Basic 1 (*M*_4_, direct)	−0.0008	0.0015	yes
Basic 2 (*M*_1_, total)	−366.75	0.2025	no
Basic 2 (*M*_2_, direct)	−0.0098	0.1754	yes
Basic 2 (*M*_3_, total)	1.2676	0.0022	no
Basic 2 (*M*_4_, direct)	−0.0008	0.0015	yes
Basic 3 (*M*_1_, total)	−100.24	0.2025	no
Basic 3 (*M*_2_, direct)	−100.02	0.1441	no
Basic 3 (*M*_3_, total)	0.3283	0.0025	no
Basic 3 (*M*_4_, direct)	0.5611	0.0014	no
Basic 4 (*M*_1_, total)	−466.75	0.2025	no
Basic 4 (*M*_2_, direct)	−100.01	0.1754	no
Basic 4 (*M*_3_, total)	1.6370	0.0021	no
Basic 4 (*M*_4_, direct)	0.5611	0.0014	no

Legend. Mean of the estimated exposure (ICS) effect (mean difference or log odds ratio) on the outcome (BW, LBW or SGA) based on 1000 samples of size 20,000, with Monte Carlo standard error (se)

Basic Scenario 1: no ICS effect on BW; Basic Scenario 2: indirect ICS effect on BW;

Basic Scenario 3: direct ICS effect on BW; Basic Scenario 4: direct and indirect ICS effect on BW

*M*_1_: *BW* ~ *ICS*; *M*_2_: *BW* ~ *ICS* + *GA*; *M*_3_: *LBW* ~ *ICS*; *M*_4_: *SGA* ~ *ICS*;

null ICS effect = yes if 0 ∈ mean ± 1.96se

*Abbreviations*: *BW* birth weight, *GA* gestational age, *ICS* inhaled corticosteroids, *LBW* low birth weight, *SGA* small for gestational age

**Table 4:** Confounding scenarios for indirect ICS effect on BW

Scenario (model, interpretation of effect)	mean	se	null ICS effect?	association confounded?
Confounding 1 (*M*_1_, total)	−513.25	0.2041	no	yes
Confounding 1 (*M*_1,*V*_, total)	−366.72	0.2189	no	no
Confounding 1 (*M*_2_, direct)	−0.1645	0.1814	yes	no
Confounding 1 (*M*_2,*V*_, direct)	−0.1614	0.1823	yes	no
Confounding 1 (*M*_3_, total)	1.7763	0.0017	no	yes
Confounding 1 (*M*_3,*V*_, total)	1.3296	0.0018	no	no
Confounding 1 (*M*_4_, direct)	−0.0011	0.0015	yes	no
Confounding 1 (*M*_4,*V*_, direct)	−0.0001	0.0016	yes	no
Confounding 2 (*M*_1_, total)	−366.44	0.2292	no	no
Confounding 2 (*M*_1,*V*_, total)	−366.56	0.1973	no	no
Confounding 2 (*M*_2_, direct)	24.232	0.1690	no	yes
Confounding 2 (*M*_2,*V*_, direct)	−0.1271	0.1694	yes	no
Confounding 2 (*M*_3_, total)	1.2508	0.0014	no	no
Confounding 2 (*M*_3,*V*_, total)	1.3351	0.0014	no	no
Confounding 2 (*M*_4_, direct)	0.0008	0.0013	yes	no
Confounding 2 (*M*_4,*V*_, direct)	0.0010	0.0013	yes	no
Confounding 3 (*M*_1_, total)	−406.67	0.1950	no	yes
Confounding 3 (*M*_1,*V*_, total)	−366.72	0.2189	no	no
Confounding 3 (*M*_2_, direct)	−40.136	0.1714	no	yes
Confounding 3 (*M*_2,*V*_, direct)	−0.1614	0.1823	yes	no
Confounding 3 (*M*_3_, total)	1.4315	0.0021	no	yes
Confounding 3 (*M*_3,*V*_, total)	1.2931	0.0023	no	no
Confounding 3 (*M*_4_, direct)	0.2212	0.0013	no	yes
Confounding 3 (*M*_4,*V*_, direct)	0.0006	0.0014	yes	no
Confounding 4 (*M*_1_, total)	−553.24	0.2109	no	yes
Confounding 4 (*M*_1,*V*_, total)	−366.72	0.2189	no	no
Confounding 4 (*M*_2_, direct)	−10.382	0.1854	no	yes
Confounding 4 (*M*_2,*V*_, direct)	−0.1614	0.1823	yes	no
Confounding 4 (*M*_3_, total)	1.8558	0.0016	no	yes
Confounding 4 (*M*_3,*V*_, total)	1.3321	0.0016	no	no
Confounding 4 (*M*_4_, direct)	0.2212	0.0013	no	yes
Confounding 4 (*M*_4,*V*_, direct)	0.0006	0.0014	yes	no

Legend. Mean of the estimated exposure (ICS) effect (mean difference or log odds ratio) on the outcome (BW, LBW or SGA) based on 1000 samples of size 20,000, with Monte Carlo standard error (se)

Confounding Scenario 1: confounder between ICS and GA; Confounding Scenario 2: confounder between GA and BW; Confounding Scenario 3: confounder between ICS and BW; Confounding Scenario 4: confounder between ICS, GA and BW

*M*_1_: *BW ~ ICS*; *M*_2_: *BW ~ ICS + GA*; *M*_3_: *LBW ~ ICS*; *M*_4_: *SGA ~ ICS*;

null ICS effect = yes if 0 ∈ mean ± 1.96se

Models subscripted by V additionally include the V variable in the regression model

*Abbreviations*: *BW* birth weight, *GA* gestational age, *ICS* inhaled corticosteroids, *LBW* low birth weight, *SGA* small for gestational age

For Basic Scenario 1 (Table 3), all mean estimated ICS effects on (L)BW or SGA are close to zero, as is expected since ICS has no effect, either direct or indirect, on BW. For Basic Scenario 2, in which ICS has an indirect effect on BW, the ICS effect in the GA-unadjusted models *M*_1_ and *M*_3_ is significantly different from zero. In model *M*_1_, this mean estimate reflects the diminution of 2 weeks in GA for babies exposed to ICS, which in turn decreases the average BW by (−2) × 183.25 = −366.50 g (see Eq. (1)). Further, we observe that ICS is not associated with SGA (*M*_4_), nor is it with BW when we condition on GA (*M*_2_). For Basic Scenario 3, all mean estimated ICS effects on (L)BW or SGA are significantly different from zero. The mean estimated ICS effect on BW is close to −100 in both models *M*_1_ and *M*_2_, and reflects the direct effect of ICS on BW (see Eq. (1)). In that scenario, being exposed to ICS was found to increase the odds of having a LBW baby by exp (0.3283) = 1.3886 and the odds of having a SGA baby by exp (0.5611) = 1.7526. In Basic Scenarios 1 and 3, a reduction in variability is observed for the ICS estimates obtained from model *M*_2_ as opposed to model *M*_1._ In these scenarios, the mean estimated ICS effect is the same for both models *M*_1_ and *M*_2,_ but the effect is more accurately estimated from the model including GA (*M*_2_). For Basic Scenario 4, the mean estimated ICS effect on BW in *M*_1_ and *M*_2_ are different, as in Basic Scenario 2. In this case, the estimated ICS effect on BW in *M*_1_ represents both the direct and indirect effects specified in simulation (−100–366.50 = −466.50 g), while the ICS effect on BW observed from *M*_2_ only reflects the direct effect. The estimated effect of ICS on SGA in Basic Scenario 4 is the same as in Basic Scenario 3. Together, the pairs of Basic Scenarios (1, 2) and Basic Scenarios (3, 4) thus reveal that the strength of the association between the exposure and SGA is driven by the size of the direct effect of the exposure on BW.

For interpreting the results from the confounding scenarios (Table 4), we take as reference the results obtained for Basic Scenario 2. In Confounding Scenario 1, in which V is a confounder between ICS and GA, only the estimated ICS effects from model *M*_1_ and *M*_3_ are biased. These results are interesting since they show that common causes of exposure and GA, and not only common causes of exposure and BW, create bias in the exposure-BW relationship when the (L)BW model do not adjust for them. In such a fully mediated scenario, this situation arises because of the presence of the open back-door path *ICS* ← *V* → *GA* → *BW* which creates a spurious association between ICS and BW; this path becomes closed when conditioning on GA. Still in Confounding Scenario 1, the mean estimate in model *M*_3,*V*_ (1.3296) is somewhat different from the mean estimate for *M*_3_ in the Basic Scenario 2 (1.2676). This difference is attributed to the well-known non-collapsibility of the odds ratios, where, on this scale, marginal effects are generally different than conditional effects [25]. In Confounding Scenario 2, in which V is a confounder between GA and BW, the estimated ICS effects from model *M*_2_ are biased. This bias arises since adjusting for GA in the BW model opens the back-door path *ICS* → *GA* ← *V* → *BW* which creates a spurious association between ICS and BW; including V in the model closes the path and thus eliminates the bias (see corresponding result for model *M*_2,*V*_). In Confounding Scenario 3, in which V is a confounder between ICS and BW, all estimated ICS effects from non V-adjusted models (i.e., models *M*_1_, *M*_2_, *M*_3,_ *M*_4_) are biased. Without surprise, all estimated ICS effects from non V-adjusted models are also biased in Confounding Scenario 4 (V confounder for all ICS, GA, and BW). Overall, there are thus two confounding scenarios that affect the estimates differently depending on whether we use (L)BW or SGA as outcome variables. Unlike (L)BW, our results indicate that SGA is not affected by confounders between the exposure and GA, nor by those between GA and BW. In particular, it appears that using this outcome variable prevents the collider bias problem that was seen under model *M*_2_ for the conditional effect of ICS on BW in Confounding Scenario 2.

### Sensitivity analysis results

The results for the sensitivity analyses regarding the heterogeneity of errors in basic and confounding scenarios are found in Tables 5 and 6, respectively.

**Table 5:** Basic scenarios without confounding with heterogeneity of errors

Scenario (model, interpretation of effect)	mean	se	null ICS effect?
Basic 1 (*M*_1_, total)	−0.2350	0.2398	yes
Basic 1 (*M*_2_, direct)	−0.0126	0.1931	yes
Basic 1 (*M*_3_, total)	0.0029	0.0022	yes
Basic 1 (*M*_4_, direct)	−0.0008	0.0015	yes
Basic 2 (*M*_1_, total)	−366.75	0.2388	no
Basic 2 (*M*_2_, direct)	−0.0374	0.2258	yes
Basic 2 (*M*_3_, total)	1.1826	0.0018	no
Basic 2 (*M*_4_, direct)	−0.0008	0.0015	yes
Basic 3 (*M*_1_, total)	−100.24	0.2398	no
Basic 3 (*M*_2_, direct)	−100.01	0.1931	no
Basic 3 (*M*_3_, total)	0.3250	0.0020	no
Basic 3 (*M*_4_, direct)	0.4236	0.0014	no
Basic 4 (*M*_1_, total)	−466.75	0.2388	no
Basic 4 (*M*_2_, direct)	−100.04	0.2258	no
Basic 4 (*M*_3_, total)	1.5032	0.0018	no
Basic 4 (*M*_4_, direct)	0.4316	0.0014	no

Legend. Mean of the estimated exposure (ICS) effect (mean difference or log odds ratio) on the outcome (BW, LBW or SGA) based on 1000 samples of size 20,000, with Monte Carlo standard error (se)

Basic Scenario 1: no ICS effect on BW; Basic Scenario 2: indirect ICS effect on BW;

Basic Scenario 3: direct ICS effect on BW; Basic Scenario 4: direct and indirect ICS effect on BW

*M*_1_: *BW* ~ *ICS*; *M*_2_: *BW* ~ *ICS* + *GA*; *M*_3_: *LBW* ~ *ICS*; *M*_4_: *SGA* ~ *ICS*;

null ICS effect = yes if 0 ∈ mean ± 1.96se

*Abbreviations*: *BW* birth weight, *GA* gestational age, *ICS* inhaled corticosteroids, *LBW* low birth weight, *SGA* small for gestational age

**Table 6:** Confounding scenarios for indirect ICS effect on BW with heterogeneity of errors

Scenario (model, interpretation of effect)	mean	se	null ICS effect?
Confounding 1 (*M*_1_, total)	−513.28	0.2388	no
Confounding 1 (*M*_1,*V*_, total)	−366.76	0.2579	no
Confounding 1 (*M*_2_, direct)	−0.2363	0.2328	yes
Confounding 1 (*M*_2,*V*_, direct)	−0.2306	0.2353	yes
Confounding 1 (*M*_3_, total)	1.5614	0.0015	no
Confounding 1 (*M*_3,*V*_, total)	1.1601	0.0016	no
Confounding 1 (*M*_4_, direct)	−0.0011	0.0015	yes
Confounding 1 (*M*_4,*V*_, direct)	−0.0001	0.0016	yes
Confounding 2 (*M*_1_, total)	−366.46	0.2600	no
Confounding 2 (*M*_1,*V*_, total)	−366.58	0.2312	no
Confounding 2 (*M*_2_, direct)	24.188	0.2168	no
Confounding 2 (*M*_2,*V*_, direct)	−0.1823	0.2167	yes
Confounding 2 (*M*_3_, total)	1.0759	0.0012	no
Confounding 2 (*M*_3,*V*_, total)	1.1475	0.0012	no
Confounding 2 (*M*_4_, direct)	0.0125	0.0013	no
Confounding 2 (*M*_4,*V*_, direct)	0.0127	0.0013	no
Confounding 3 (*M*_1_, total)	−406.71	0.2335	no
Confounding 3 (*M*_1,*V*_, total)	−366.76	0.2593	no
Confounding 3 (*M*_2_, direct)	−40.210	0.2226	no
Confounding 3 (*M*_2,*V*_, direct)	−0.2394	0.2378	yes
Confounding 3 (*M*_3_, total)	1.3095	0.0017	no
Confounding 3 (*M*_3,*V*_, total)	1.1839	0.0019	no
Confounding 3 (*M*_4_, direct)	0.1738	0.0014	no
Confounding 3 (*M*_4,*V*_, direct)	0.0050	0.0016	no
Confounding 4 (*M*_1_, total)	−553.28	0.2444	no
Confounding 4 (*M*_1,*V*_, total)	−366.76	0.2579	no
Confounding 4 (*M*_2_, direct)	−10.454	0.2361	no
Confounding 4 (*M*_2,*V*_, direct)	−0.2306	0.2353	yes
Confounding 4 (*M*_3_, total)	1.6301	0.0013	no
Confounding 4 (*M*_3,*V*_, total)	1.1518	0.0014	no
Confounding 4 (*M*_4_, direct)	0.1859	0.0014	no
Confounding 4 (*M*_4,*V*_, direct)	0.0120	0.0015	no

Legend. Mean of the estimated exposure (ICS) effect (mean difference or log odds ratio) on the outcome (BW, LBW or SGA) based on 1000 samples of size 20,000, with Monte Carlo standard error (se)

Confounding Scenario 1: confounder between ICS and GA; Confounding Scenario 2: confounder between GA and BW; Confounding Scenario 3: confounder between ICS and BW; Confounding Scenario 4: confounder between ICS, GA and BW

*M*_1_: *BW ~ ICS; M*_2_: *BW ~ ICS + GA; M*_*3*_: *LBW ~ ICS;* M_4_: *SGA ~ ICS*;

null ICS effect = yes if 0 ∈ mean ± 1.96se

Models subscripted by V additionally include the V variable in the regression model

*Abbreviations*: *BW* birth weight, *GA* gestational age, *ICS* inhaled corticosteroids, *LBW* low birth weight, *SGA* small for gestational age

In the basic scenarios with heterogeneity of errors (Table 5), the same interpretation as in the basic scenarios with homogeneity of errors can be done regarding the type of effect (total, direct) estimated in the different (L)BW and SGA models.

In the confounding scenarios with heterogeneity of errors (Table 6), the estimates obtained behaved similarly to those obtained under the confounding scenarios with homogeneity of errors from one scenario to the other. One notable difference is with regard to the non-null effect for some SGA models in Confounding Scenarios 2–4. Specifically, Models *M*_4_, *M*_4,*V*_ in Confounding Scenarios 2 and *M*_4,*V*_ in Confounding Scenarios 3 and 4 all featured a mean ICS estimate very close but significantly different from zero. For example, the mean estimate from *M*_4,*V*_ in Confounding Scenarios 2 with heterogeneity of error was 0.0127 and the standard error of the mean was 0.0013, thus only slightly, but significantly, departing from the null. This is opposed to the null effect found in the corresponding *M*_4,*V*_ result when assuming homogeneity of errors. A common feature of Confounding Scenarios 2–4, which could be the source of this very small discrepancy in these fully mediated scenarios, is the GA-dependent heterogeneity of effect of V on SGA. Indeed, in these scenarios, the direct effect of the V covariate on the mean BW is non-null and the same for all GA values (−100); however, when assuming heterogeneity of errors, this effect will translate differently across GA for SGA since the BW standard deviation, which intervenes in the definition of the SGA variable, varies across GA.

The results for the sensitivity analyses regarding the nonlinear GA effect on BW in basic and confounding scenarios are found in Tables 7 and 8.

**Table 7:** Basic scenarios without confounding with nonlinear GA effect on BW

Scenario (model, interpretation of effect)	mean	se	null ICS effect?
Basic 1 (*M*_1_, total)	−0.2921	0.2050	yes
Basic 1 (*M*_2_, direct)	−0.0693	0.1479	yes
Basic 1 (${M}_2^{\ast }$, direct)	−0.0137	0.1442	yes
Basic 1 (*M*_3_, total)	0.0049	0.0026	yes
Basic 1 (*M*_4_, direct)	−0.0008	0.0015	yes
Basic 2 (*M*_1_, total)	−359.88	0.2132	no
Basic 2 (*M*_2_, direct)	40.140	0.1773	no
Basic 2 (${M}_2^{\ast }$, direct)	−0.0502	0.1856	yes
Basic 2 (*M*_3_, total)	1.3082	0.0021	no
Basic 2 (*M*_4_, direct)	−0.0008	0.0015	yes
Basic 3 (*M*_1_, total)	−100.29	0.2050	no
Basic 3 (*M*_2_, direct)	−100.07	0.1479	no
Basic 3 (${M}_2^{\ast }$, direct)	−100.01	0.1442	no
Basic 3 (*M*_3_, total)	0.2730	0.0024	no
Basic 3 (*M*_4_, direct)	0.5611	0.0014	no
Basic 4 (*M*_1_, total)	−459.88	0.2132	no
Basic 4 (*M*_2_, direct)	−59.860	0.1773	no
Basic 4 (${M}_2^{\ast }$, direct)	−100.05	0.1856	no
Basic 4 (*M*_3_, total)	1.6259	0.0020	no
Basic 4 (*M*_4_, direct)	0.5611	0.0014	no

Legend. Mean of the estimated exposure (ICS) effect (mean difference or log odds ratio) on the outcome (BW, LBW or SGA) based on 1000 samples of size 20,000, with Monte Carlo standard error (se)

Basic Scenario 1: no ICS effect on BW; Basic Scenario 2: indirect ICS effect on BW;

Basic Scenario 3: direct ICS effect on BW; Basic Scenario 4: direct and indirect ICS effect on BW

*M*_1_: *BW* ~ *ICS*; *M*_2_: *BW* ~ *ICS* + *GA*; ${M}_2^{\ast }$: *BW ~ ICS + GA* + *GA* ^2^ + *GA* ^3^; *M*_3_: *LBW* ~ *ICS*; *M*_4_: *SGA* ~ *ICS*;

null ICS effect = yes if 0 ∈ mean ± 1.96se

*Abbreviations*: *BW* birth weight, *GA* gestational age, *ICS* inhaled corticosteroids, *LBW* low birth weight, *SGA* small for gestational age

**Table 8:** Confounding scenarios for indirect ICS effect on BW simulated with nonlinear GA effect on BW

Scenario (model, interpretation of effect)	mean	se	null ICS effect?
Confounding 1 (*M*_1_, total)	−560.30	0.2198	no
Confounding 1 (*M*_1,*V*_, total)	−400.33	0.2377	no
Confounding 1 (*M*_2_, direct)	16.904	0.1866	no
Confounding 1 (*M*_2,*V*_, direct)	15.329	0.1869	no
Confounding 1 (${M}_2^{\ast }$, direct)	−0.1993	0.1916	yes
Confounding 1 (${M}_{2,V}^{\ast }$, direct)	−0.1992	0.1917	yes
Confounding 1 (*M*_3_, total)	1.9051	0.0017	no
Confounding 1 (*M*_3,*V*_, total)	1.4397	0.0017	no
Confounding 1 (*M*_4_, direct)	−0.0011	0.0015	yes
Confounding 1 (*M*_4,*V*_, direct)	−0.0001	0.0016	yes
Confounding 2 (*M*_1_, total)	−400.02	0.2474	no
Confounding 2 (*M*_1,*V*_, total)	−400.15	0.2139	no
Confounding 2 (*M*_2_, direct)	39.335	0.1709	no
Confounding 2 (*M*_2,*V*_, direct)	19.839	0.1745	no
Confounding 2 (${M}_2^{\ast }$, direct)	31.644	0.1712	no
Confounding 2 (${M}_{2,V}^{\ast }$, direct)	−0.1679	0.1774	yes
Confounding 2 (*M*_3_, total)	1.3528	0.0013	no
Confounding 2 (*M*_3,*V*_, total)	1.4636	0.0014	no
Confounding 2 (*M*_4_, direct)	0.0008	0.0013	yes
Confounding 2 (*M*_4,*V*_, direct)	0.0010	0.0013	yes
Confounding 3 (*M*_1_, total)	−399.77	0.2051	no
Confounding 3 (*M*_1,*V*_, total)	−359.81	0.2317	no
Confounding 3 (*M*_2_, direct)	−0.0345	0.1714	no
Confounding 3 (*M*_2,*V*_, direct)	39.950	0.1846	no
Confounding 3 (${M}_2^{\ast }$, direct)	−40.216	0.1852	no
Confounding 3 (${M}_{2,V}^{\ast }$, direct)	−0.2350	0.1955	yes
Confounding 3 (*M*_3_, total)	1.4528	0.0021	no
Confounding 3 (*M*_3,*V*_, total)	1.3339	0.0023	no
Confounding 3 (*M*_4_, direct)	0.2212	0.0013	no
Confounding 3 (*M*_4,*V*_, direct)	0.0006	0.0014	yes
Confounding 4 (*M*_1_, total)	−600.29	0.2267	no
Confounding 4 (*M*_1,*V*_, total)	−400.33	0.2377	no
Confounding 4 (*M*_2_, direct)	6.6866	0.1907	no
Confounding 4 (*M*_2,*V*_, direct)	15.329	0.1869	no
Confounding 4 (${M}_2^{\ast }$, direct)	−1.6285	0.1930	no
Confounding 4 (${M}_{2,V}^{\ast }$, direct)	−0.1992	0.1917	yes
Confounding 4 (*M*_3_, total)	1.9757	0.0015	no
Confounding 4 (*M*_3,*V*_, total)	1.4531	0.0016	no
Confounding 4 (*M*_4_, direct)	0.2212	0.0013	no
Confounding 4 (*M*_4,*V*_, direct)	0.0006	0.0014	yes

Legend. Mean of the estimated exposure (ICS) effect (mean difference or log odds ratio) on the outcome (BW, LBW or SGA) based on 1000 samples of size 20,000, with Monte Carlo standard error (se)

Confounding Scenario 1: confounder between ICS and GA; Confounding Scenario 2: confounder between GA and BW; Confounding Scenario 3: confounder between ICS and BW; Confounding Scenario 4: confounder between ICS, GA and BW

*M*_1_ *: BW ~ ICS; M*_2_: *BW ~ ICS + GA;* ${M}_2^{\ast }$: *BW ~ ICS + GA* + *GA* ^2^ + *GA* ^3^ *; M*_3_: *LBW ~ ICS; M*_4_: *SGA ~ ICS*;

null ICS effect = yes if 0 ∈ mean ± 1.96se

Models subscripted by V additionally include the V variable in the regression model.

*Abbreviations*: *BW* birth weight, *GA* gestational age, *ICS* inhaled corticosteroids, *LBW* low birth weight, *SGA* small for gestational age

With a nonlinear GA effect on BW, changes in results were observed for Basic Scenarios 2 and 4 which both feature an indirect effect of GA on BW (Table 7). For these scenarios, the effect obtained under model *M*_2_, which adjusts for a linear GA term only, did not unbiasedly represent the direct effect of exposure on BW. A positive residual bias of about 40 g was observed, but this bias vanished when additionally including the quadratic and cubic GA terms in the model (see results for ${M}_2^{\ast }$ in these scenarios).

Compared to all previous scenarios, the interpretation of results for the confounding scenarios under nonlinearity of GA effect on BW is more complicated (Table 8). It is noted that even BW models adjusted for V (*M*_1,*V*_) did not estimate the total effect of exposure completely without bias when the confounder was associated with GA (Confounding Scenarios 1, 2, 4). However, model *M*_1,*V*_ did unbiasedly estimate the total effect of exposure under Confounding Scenarios 3 (refer to *M*_1,*V*_ in Basic Scenario 2, Table 7, for comparison), scenario in which the confounder is associated with exposure and outcome only. Nonetheless, adjusting for V in the (L)BW models *M*_1,*V*_ and *M*_3,*V*_ yielded estimates overall closer to the total effect of exposure, as expected. The unbiased estimation of the direct effect of exposure in the BW models was achieved under model ${M}_{2,V}^{\ast }$, where both the correct functional form of GA and the confounder was accounted for. In these sensitivity analyses, no changes in results and interpretation were observed for SGA as compared with the Confounding Scenarios in the primary analyses (Table 4).

## Discussion

Our study found that the sole consideration of SGA in a study may lead to suboptimal understanding and quantification of the underlying effect of an exposure on BW-related measures. Using DAGs where GA was a potential mediator between the exposure and BW, we have confirmed that SGA is an absorbing variable: the observed association between the exposure and SGA solely reflects the direct effect of the exposure on BW, effect which could be interpreted as a manifestation of intrauterine growth retardation. In the situation where the effect of exposure on BW was fully mediated by GA, the exposure and SGA were *not* causally linked. Therefore, an analyst may have concluded for no exposure effect on the BW-axis, but what should really be concluded is the absence of a *direct effect* of the exposure on BW. While one could argue this is precisely the purpose of using SGA, we believe that being able to assess the total (direct and indirect) effect of the exposure on BW is at least as valuable.

If the exposure has a direct beneficial effect but an overall detrimental effect, the direct effect has less importance. However, precise quantification of the total effect is obtained with the use of BW as outcome variable. From our perspective, it would thus not be advisable to only consider SGA and GA as outcome variables since the ability to precisely assess the indirect and total effects of exposure on BW would be diminished. Although the presence of an indirect effect of the exposure on BW could be deduced from results looking at the effect of the exposure on GA, our study suggests that considering BW as outcome variable is the most straightforward way to thoroughly investigate this issue. Indeed, recall that the strength of an indirect effect of an exposure on an outcome is a combination of two measures: 1) the strength of the association between the exposure and the mediator (GA) and 2) the strength of the association between the mediator and the outcome (BW) [26].

One advantage we found with respect to the use of SGA as outcome variable, as opposed to BW, is that is less prone to bias. Interestingly, exposure effect estimates from SGA models were found unbiased whenever exposure effect estimates from BW models were affected by collider bias due to conditioning on GA in the model. Therefore, accounting for GA internally (through the use of a GA-adjusted BW measure) or externally (by conditioning on GA in a model for BW) are two competing strategies that are not equally robust to bias. This finding is particularly important when confounders for the GA and BW association are unmeasured and cannot be adjusted for in the model for BW. In our study, only the SGA model, and not the BW model, yielded unbiased results interpretable as a direct effect of exposure on BW in the absence of such a confounder in the model. We also found that whenever one missing covariate was a common cause between exposure and BW, the exposure-BW association as well as the exposure-SGA association were biased. Therefore, one should not make the distinction between these outcome variables when selecting such confounders and adjusting for them in models. However, we found that in fully mediated sets-up, common causes between exposure and GA biased the exposure-(L)BW relationship unlike the exposure-SGA relationship when a model with the former variable (BW) did not adjust for GA. Distinguishing between common causes of exposure and GA and common causes of exposure and (L)BW could, however, be a rather difficult task in practice.

## Conclusions

In light of our simulations and current wisdom, we recommend that, in addition to GA, both outcome variables (L)BW and SGA be considered in studies that rely on these perinatal outcomes. Alternatively to considering all three outcomes (GA, SGA, and (L)BW) in standard separate analyses, mediation analyses with GA as a mediator could be used to better understand the direct and indirect effects of an exposure on BW. When adopting a mediation strategy, the use of SGA could be omitted as one would be able to make the distinction between an exposure effect on (L)BW arising through a diminution in GA and one external to this mechanism. However, such a mediation model would nevertheless be inadequate to provide unbiased results in the presence of unmeasured mediator-outcome confounders [11] and could also be sensitive to linearity assumptions.

Globally, our study has highlighted the complexity of perinatal outcome modeling. Although our findings are directly relevant to the field of maternal asthma, we believe they are applicable to other research areas or specific types of studies where (L)BW and SGA, as measures of perinatal health, are the most useful and feasible. As a matter of fact, these variables have been recently considered in meta-analyses (e.g., [27]), large clinical trials in less-resourced countries (e.g., [28]), and large-scale perinatal studies based on administrative databases (e.g., [29]).

## Abbreviations

BW: Birth weight
DAG: Directed acyclic graph
GA: Gestational age
ICS: Inhaled corticosteroids
LBW: Low birth weight
PTB: Preterm birth
SGA: Small for gestational age
