# Correction to: On the use of the outcome variable “small for gestational age” when gestational age is a potential mediator: a maternal asthma perspective

**DOI:** 10.1186/s12874-018-0477-y

**Published:** 2018-01-29

**Authors:** Geneviève Lefebvre, Mariia Samoilenko

**Affiliations:** 1 Department of Mathematics, Université du Québec à Montréal, C.P. 8888, Succursale Centre-ville, Montréal, Québec H3C 3P8 Canada; 2 Faculty of Pharmacy, Université de Montréal, Montréal, Canada

## Correction

Following publication of the original article [1], the authors reported that the following four references inTable 2 were incorrect:

Lao et al. [15]^b^

Braken et al. [23]^b^

Bakhireva et al. [26]^b^

Clark et al. [28]^b^

These references have already been introduced in Table 1 and should read:

Lao et al. [30]

Bracken et al. [38]

Bakhireva et al. [41]

Clark et al. [43]

The original article has been updated.
